# Supplementary material for: High Incidence of Candidemia in Critically Ill COVID-19 Patients Supported by Veno-Venous Extracorporeal Membrane Oxygenation: A Retrospective Study
Source: J Fungi (Basel). 2023 Jan 14;9(1):119. doi: 10.3390/jof9010119 (PMC9861971; doi:10.3390/jof9010119)
Supplement: Supplementary file 1 [file jof-09-00119-s001.zip › jof-2153268-supplementary.pdf]

Supplementary Table S1: Multivariable competing risk Fine Gray regression model for candidaemia without Propensity Score (among the patients admittede to the intensive care unit of Policlinico Umberto I teaching hospital of rome between March2020 and June 2021 and who underwent intubation and mechanical ventilation).

	**Candidaemia**	
	**aSHR (95% CI)**	***p*-value**
ECMO support	2.31 (1.15-4.64)	0.019
Non-candidaemic BSI	1.61 (0.22-1.63)	0.353
Tocilizumab therapy	0.60 (0.22-1.63)	0.319
Inotropes Therapy	0.97 (0.29-3.18)	0.957
Vasopressor Therapy	0.19 (0.08-0.44)	<0.001
Candida score	2.88 (2.19-3.79)	<0.001

aSHR: Adjusted Subdistribution Hazard Ratio; CI: Confidence Interval. ECMO: Extracorporeal Membrane Oxygenation; BSI: Blood Stream Infection; Ref; reference

Supplementary Table S2. Multivariable logistic regression models for candidaemia stratified on ECMO support (among the patients admitted to the intensive care unit of Umberto I teaching hospital of Rome between March 2020 and June 2021 and who underwent IOT and mechanical ventilation)

	Candidaemia			
	ECMO support		No ECMO support	
	**aSHR (95% CI)**	***p*-value**	**aSHR (95% CI)**	***p*-value**
Age				
<62 years	Ref.		Ref.	
>62 years	0.91 (0.80-1.04)	0.171	0.97 (0.92-1.03)	0.355
Gender				
Male	Ref.		Ref.	
Female	0.33 (0.20-5.34)	0.433	2.71 (0.69-10.61)	0.152
Non-candidaemic BSI	0.02 (0.01-3.42)	0.135	4.94 (1.22-20.02)	0.025
Candida score	27.43 (1.79-419.98)	0.017	2.14 (1.14-10.61)	0.017
ECMO length, days	0.94 (0.80-1.11)	0.474	--	--

aSHR: Adjusted Subdistribution Hazard Ratio; CI: Confidence Interval. ECMO: Extracorporeal Membrane Oxygenation; BSI: Blood Stream Infection; Ref; reference.
